# Analysis of Acute and Short-Term Fluoride Toxicity in Zebrafish Embryo and Sac–Fry Stages Based on Bayesian Model Averaging

**DOI:** 10.3390/toxics12120902

**Published:** 2024-12-11

**Authors:** Tingxu Jin, Xiumei Yang, Yuanhui Zhu, Cheng Yan, Rui Yan, Qianlei Yang, Hairu Huang, Yan An

**Affiliations:** 1 Department of Toxicology, School of Public Health, Jiangsu Key Laboratory of Preventive and Translational Medicine for Major Chronic Non-communicable Diseases, MOE Key Laboratory of Geriatric Diseases and Immunology, Suzhou Medical College of Soochow University, Suzhou 215123, China; zbzyhcom@163.com (Y.Z.); 15895572995@163.com (R.Y.); qlyang@suda.edu.cn (Q.Y.); 20214247033@stu.suda.edu.cn (H.H.); 2 School of Public Health, The Key Laboratory of Environmental Pollution Monitoring and Disease Control, Ministry of Education, Guizhou Medical University, No.6 Ankang Road, Guian New Area, Guizhou 561113, China; 2023110040831@stu.gmc.edu.cn; 3 School of Environmental Studies, China University of Geosciences, Wuhan 430074, China; cheng_yan@cug.edu.cn

**Keywords:** water fluoride concentration, early life stage, aquatic toxicology, LC_50_, EC_50_, Bayesian BMC

## Abstract

Acute and short-term toxicity tests are foundational to toxicology research. These tests offer preliminary insights into the fundamental toxicity characteristics of the chemicals under evaluation and provide essential data for chronic toxicity assessments. Fluoride is a common chemical in aquatic environments; however, the findings of toxicological data, such as LC_50_ for aquatic organisms, often exhibit inconsistency. Consequently, this study employed zebrafish as a model organism during their early life stages to assess the acute and short-term toxicity of fluoride exposure. Bayesian model averaging was utilized to calculate the LC_50_/EC_50_ values and establish baseline concentrations. The results indicated a dose–response relationship between water fluoride concentration and harmful outcomes. The 20 mg/L group was identified as the lowest observed adverse effect level (LOAEL) for the majority of toxicity indicators and warrants special attention. Based on the BBMD model averages, the LC_50_ of fluoride for 1 to 5 days post-fertilization (dpf) zebrafish was 147.00, 80.80, 61.25, 56.50, and 37.50 mg/L, while the EC_50_ of cumulative malformation rate for 5 dpf zebrafish was 59.75 mg/L. As the benchmark response (BMR) increased, both the benchmark concentrations (BMCs) and benchmark dose levels (BMDLs) also increased. The research aims to provide essential data for the development of environmental water guidelines and to mitigate ecological risks associated with fluoride in aquatic ecosystems.

## 1. Introduction

Acute and short-term toxicity tests are fundamental components of toxicology research. These tests provide essential toxicological data regarding the test chemical, including median lethal dose (LC_50_), effect concentration for 50% effect (EC_50_), No Observed Adverse Effect Level (NOAEL), and Lowest observed adverse effect level (LOAEL). The acute toxicity test is specifically designed to determine the lethal dose or concentration of the test chemical in experimental animals, offering a preliminary estimation of the associated toxicity risk [1,2]. Furthermore, it seeks to elucidate the dose–response relationship between acute toxicity and the toxic characteristics of the test chemical [3]. The short-term toxicity test, as an extension, is designed to identify both lethal and limited sublethal effects of chemicals on specific stages and species. This test serves as a bridge between lethal and sublethal assessments, providing foundational data that support more complex investigations into sublethal effects, including physiological and behavioral changes, ecological toxicity, and the screening for chronic toxicity or whole-life stage tests [4]. The acute and short-term toxicity of chemicals is not entirely consistent across organisms at different life stages [5]. The early life stage, which encompasses the period from embryo to birth, represents a critical ‘window of opportunity’ for growth and development. Conducting acute and short-term toxicity tests during this timeframe not only yields traditional toxicological data but also facilitates the observation of stillbirths, deformities, and developmental delays induced by the tested chemicals. Furthermore, constructing dose–response models from these experimental results can provide valuable data for assessing potential ecological risks associated with the tested chemicals.

Fluoride is widely present in natural environments, being one of the strongest oxidants among known elements and the thirteenth most abundant element in the Earth’s crust [6]. The fluoride generated by both natural and anthropogenic factors constitutes the fluoride cycle within the ecosystem. The weathering of the mineral fluorite releases fluoride into the soil and groundwater [7]. Volcanic activity contributes fluoride to the soil through solidified magma following an eruption, while gas emissions containing hydrofluoric acid can contaminate the atmosphere with fluoride [8]. Additionally, marine aerosols release approximately 20,000 kg of inorganic fluoride into the atmosphere annually, with certain fluorinated gases being transported to the stratosphere [9]. These gaseous fluorides eventually settle in the soil over time [10]. Fluoride can also enter the environment through industrial and agricultural activities, as well as the discharge of domestic pollutants [11,12,13,14,15]. This fluoride subsequently enters biological organisms via various pathways, leading to a range of health risks [16,17,18,19].

Water is the most prevalent source of fluoride exposure in the environment [20]. Therefore, conducting acute and short-term toxicity experiments on fluoride exposure during the early life stages of aquatic organisms, along with calculating benchmark concentrations, can provide essential data for the formulation of environmental water guidelines and for reducing the ecological risks linked to fluoride in aquatic ecosystems. However, the findings of toxicological data, such as LC_50_ for aquatic organisms, often exhibit inconsistency, which primarily stems from the use of different experimental fish species across related studies and varying exposure durations [21,22,23,24,25]. Zebrafish are recognized as a sentinel species in aquatic environments and rank as the third most commonly used model organism in scientific research. They are endorsed as a standard model organism for chemical toxicity testing by the Organization for Economic Cooperation and Development (OECD), as well as in the Chinese technical guidelines for developing water quality standards for freshwater aquatic organisms [26,27]. Zebrafish are widely utilized in fields such as chemical toxicity assessment and ecological risk research to evaluate the potential toxicity of various compounds, including industrial wastewater, insecticides, herbicides, detergents, and pharmaceuticals [27,28,29,30]. Moreover, advancements in computational toxicology are increasing the potential for establishing human exposure thresholds based on findings from zebrafish toxicology research [31]. Consequently, conducting acute and short-term toxicity studies on fluoride exposure during the early life stages of zebrafish can provide a comprehensive understanding of fluoride toxicity and furnish essential information for updating fluoride water guidelines, thereby promoting the sustainable development of aquatic environments in the future. Furthermore, these tests are anticipated to yield fundamental data that can be used to extrapolate human exposure thresholds following technological advancements.

The LC_50_ and NOAEL of zebrafish larvae are derived from observations made during acute and short-term toxicity testing [4]. These values are determined based on the concentration of the test chemical and the corresponding mortality rate in the test animals. However, the choice of experimental design and mathematical modeling can significantly influence the results. The calculation of LC_50_ can utilize various methods, including Litchfield and Wilcoxon’s method, Karber’s method, and regression analysis. Notably, Karber’s method assumes that the response variable adheres to a normal distribution and that the exposure concentrations are organized in a proportional order [32]. Additionally, regression analysis must consider the goodness of fit for the models employed [33,34]. Litchfield and Wilcoxon’s method is widely regarded for its capacity to provide estimates of effective doses, even when data are limited. However, this strength is accompanied by a notable drawback: the method depends on the subjective placement of a straight line through hand-drawn points on graph paper. This process demands considerable time and attention, and it can yield inconsistent estimates that are vulnerable to human error [35]. The traditional NOAEL methods, as another fundamental datum, are influenced by factors including the number of experimental groups, sample size per group, and dose group spacing [32,36,37]. Consequently, the U.S. environmental protection agency (EPA) recommends utilizing the benchmark dose (BMD) and benchmark concentration (BMC), along with their respective lower limits of the 95% confidence interval (BMDL/BMCL), as substitutes [38,39]. However, selecting the appropriate model from a range of acceptable dose–response models remains a significant challenge, as common practices often fail to consider that different models may only partially represent the true dose–response relationship [40]. In order to account for uncertainty in model selection, the Bayesian model averaging (BMA) is proposed [41,42,43], which is a method to combine results from multiple models, allowing for a probabilistic interpretation of the combined cluster structure and quantification of model-based uncertainties [44]. In 2022, the European Food Safety Authority (EFSA) proposed a shift from the frequentist to the Bayesian paradigm for risk assessment. The frequentist approach measures uncertainty using confidence and significance levels, interpreted under hypothetical repetition, while the Bayesian approach assigns probability distributions to unknown parameters, extending the notion of probability to reflect the uncertainty of knowledge [45].

In summary, our study aims to calculate the LC_50_/EC_50_ and BMC/BMCL for various toxicity indicators related to water fluoride (W-F) exposure by utilizing BMA in zebrafish embryos and sac–fry stages.

## 2. Methods and Materials

### 2.1. Chemicals and Reagents

The sodium fluoride (NaF) used in this study was obtained from Zhiyuan Chemical Reagent Co., Ltd. (Tianjin, China). with a purity of 99% (CB/T: 1264-1997). Standard stock solutions of fluoride (100 mg/L) and standard solutions of fluoride (10 mg/L) were prepared following the national standard of the People’s Republic of China for water quality determination of fluoride using the ion selective electrode method (GB 7484-87) [46] by E3 water [47]. To ensure consistent concentrations throughout the research, standard stock solutions of fluoride and standard solutions of fluoride solution were stored at 4 °C, and working solutions were prepared using E3 water immediately before each experiment.

### 2.2. Selection Rationale of Fluoride Concentrations in the Current Study

To ensure that the fluoride exposure concentrations in this study reflect the potential fluoride levels present in the environment, we reviewed previous studies [21,22,23,24,25] and referenced the ‘Environmental Standard Limit Concentration’ (ESLC) for water standards to establish the fluoride exposure concentrations used in the experiment. The ESLCs used in this research were based on water environmental standards from the World Health Organization (WHO), China, and the US EPA. These standards include 0.5 mg/L [48], 1 mg/L [49,50,51,52], ≤4 mg/L [53], 10 mg/L [54], 20 mg/L [54], 50 mg/L [55], and 100 mg/L [55]; with the goal of protecting human health and aquatic organisms [56]. The final water fluoride concentrations were chosen as 0.5, 1, 4, 10, 20, 50, 80, 100, 120, 150, 200, 250 and 300 μg/L in our research. The control was E3 water.

### 2.3. Environmental Conditions for Zebrafish Experiments

Adult zebrafish were obtained from the Institute of Hydrobiology, Chinese Academy of Sciences (Wuhan, China). All fish were acclimated to their new environment for at least 2 weeks in a 50 L aquarium conditioned with water from reverse osmosis reconstituted with marine salt (Instant Ocean, Blacksburg, VA, USA) at 28 ± 2 °C, pH of 7.4, ammonia < 0.02 mg/L, nitrite < 0.01 mg/L and a conductivity of 500 μS under light/dark 14/10 photoperiod (lights on at 8:00 a.m.). The fish were used according to the OECD test guidelines No. 203 and No. 212 [2,4]. Animals were fed twice a day with live food (*Artemia salina*) and supplemented with 300 μm granules of dry feed (Haisheng Biological Experimental Equipment Co., Ltd, Shanghai, China.). The afternoon before spawning, several groups of females and males (1:1) were introduced into 1.7 L breeding tanks. Immediately after spawning, which was initiated by morning light, fertilized eggs were collected with a sieve and rinsed thoroughly with deionized water and E3 water. Eggs were transferred to Petri dishes, and eggs that were not fertilized or embryos with injuries were eliminated (30 embryos per dish with 10 cm diameter) for subsequent experiments. The Ethics Committee of the Guizhou Medical University approved the protocol under the number 2303063.

### 2.4. The Environmental Exposure of Embryo and Sac–Fry Stages Zebrafish to Fluoride

**Figure 1:**
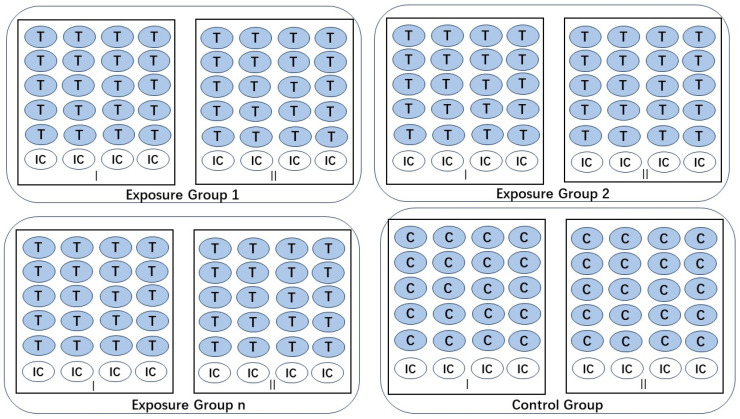
Layout of 24-well plates. T = test group; C = control group; IC = internal plate control.

To investigate the toxic effects of W-F concentrations on zebrafish embryos and sac–fry stages, zebrafish embryos were continuously exposed to different W-F concentrations from 2 h post-fertilization (hpf) (embryos) to 5 d post-fertilization (dpf) (sac–fry stages) [4]. Two parallel 24-well plates were used for each concentration group, with 40 embryos per concentration (2 embryos per well) exposed to W-F with eight internal plate controls (ICs). E3 water was used as a control group, and ICs were used for comparison. Three independent experiments were conducted, as detailed in Figure 1. The W-F solutions were changed daily, and dead embryos and larvae were removed daily.

### 2.5. Toxicological Indicators

Toxicity indicators were assessed according to OECD guidelines No. 210 and No. 212 [4,57]. The study included tracking cumulative dead numbers and cumulative mortality (CM) at concentrations on 1, 2, 3, 4, and 5 dpf, as well as cumulative numbers and cumulative malformation rate (CMA) of larvae with deformities or abnormal appearances at 5 dpf (Table 1). Observations were conducted twice daily with an 8 h interval between each observation.

### 2.6. LC/EC_50_ and BMC/BMCL Estimates

The dose–response relationship between W-F concentration and toxicity indicators were analyzed using Bayesian Benchmark Dose Analysis System (BBMD) (https://benchmarkdose.org, accessed on 28 March 2024). In order to perform the Monte Carlo approximation, a Markov chain Monte Carlo (MCMC) was constructed within the BBMD. The MCMC parameters were set as follows: MCMC iterations: 30,000; number of chains: 1; warmup fraction: 0.5; random seed: 65,323. Subsequently, the dose–response data were fitted using eight models (Table 2) in the BBMD system. The model fitting was evaluated using Rhat (1–1.05) and posterior predictive *p* value (0.05–0.95) as per the system manual [58]. The median lethal dose/effect concentration for 50% effect (LC_50_/EC_50_) of each mode was reported at the response of 50% (5th, 95th). Then, the BMC distributions estimated by each model were combined, taking into account their weights, and BMA BMC was calculated. The weight of each model was calculated based on how well it fits the data. The calculation of the BMA BMC is influenced more by models with greater weight, indicating a better overall fit. The BMC represents the dose at which there is a higher toxicological risk compared to the control group [59]. In this study, the BMCs and BMCLs for W-F were estimated given benchmark response (BMR) = 0.1 (10%).

### 2.7. Statistical Analysis

The data were analyzed using SPSS software (version 20.0, SPSS Inc., Chicago, IL, USA). Incidence data on toxicity indicators were reported as rates and the mean ± standard deviation (SD). All experimental data underwent homoscedasticity testing, and pair-wise comparisons of sample means were conducted using the least significant difference (LSD) test following a one-way analysis of variance (ANOVA). Statistical significance was determined at *p* < 0.05.

### 2.8. Quality Control and Quality Assurance

To ensure the accuracy of our study, we utilized pure water to prepare solutions of NaF and E3 water. Fluoride concentrations in all experimental groups were tested daily following the protocol outlined in GB/T 7484-87 for assessing fluoride levels in water quality [46]. It was mandated that the fluoride concentration in each group’s water should not deviate by more than 5% from the experimental design. Prior to feeding, each batch of feed was analyzed using the National Standard Method for Determination of Fluorine in Food GB/T 5009.18-2003 [60], with a requirement of less than 6.20 mg/kg.

## 3. Results

### 3.1. General Situation and Toxicological Indicators Results

**Figure 2:**
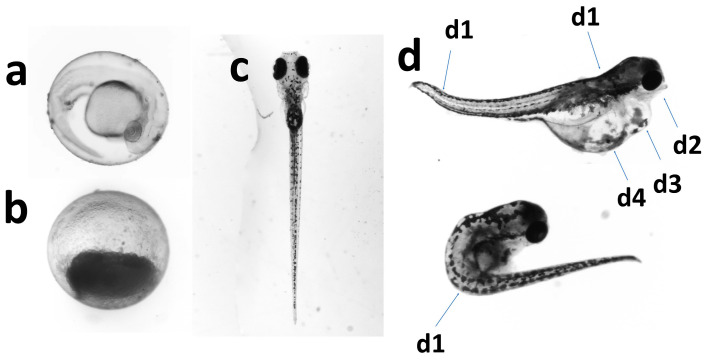
Normal and harmful outcome on embryo and sac–fry stages of zebrafish. (**a**) normal development embryo; (**b**) coagulation of the embryo; (**c**) normal larva; (**d**) malformed larva: d1, tail and spine curved; d2, mandible shorter malformation; d3, pericardial hydrops; d4, yolk sac edema.

The results indicated that as the W-F concentration increased, the CM for each day post-fertilization and CMA at 5 dpf of zebrafish embryos and larvae also increased (see Table 3). Adverse effects observed included embryo coagulation, mortality, and various types of malformations (refer to Figure 2).

**Figure 3:**
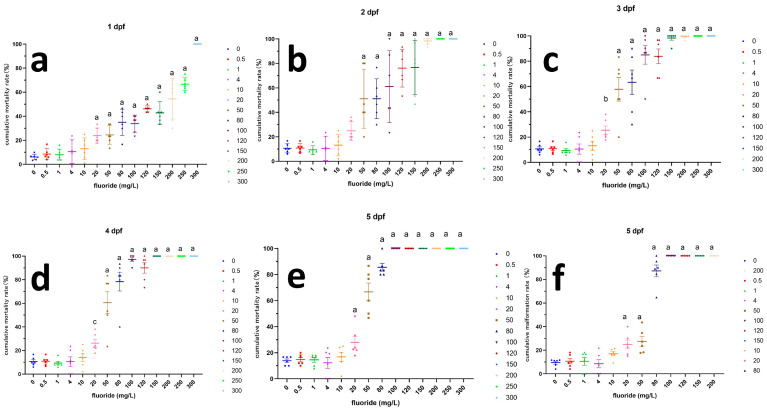
The outcome of toxicity indicators. (**a**–**e**) cumulative mortality at 1–5 dpf; (**f**) cumulative malformation rate at 5 dpf. ^a^ Compared with the control group, *p* < 0.000; ^b^ Compared with the control group, *p* < 0.05; ^c^ Compared with the control group, *p* < 0.01.

A dose–response relationship was observed between W-F concentration and harmful outcomes. The 20 mg/L group was identified as the lowest observed adverse effect level (LOAEL) for the majority of toxicity indicators and warrants special attention (Figure 3).

### 3.2. The LC_50_/EC_50_ and BMC/BMCL Estimation Results

**Figure 4:**
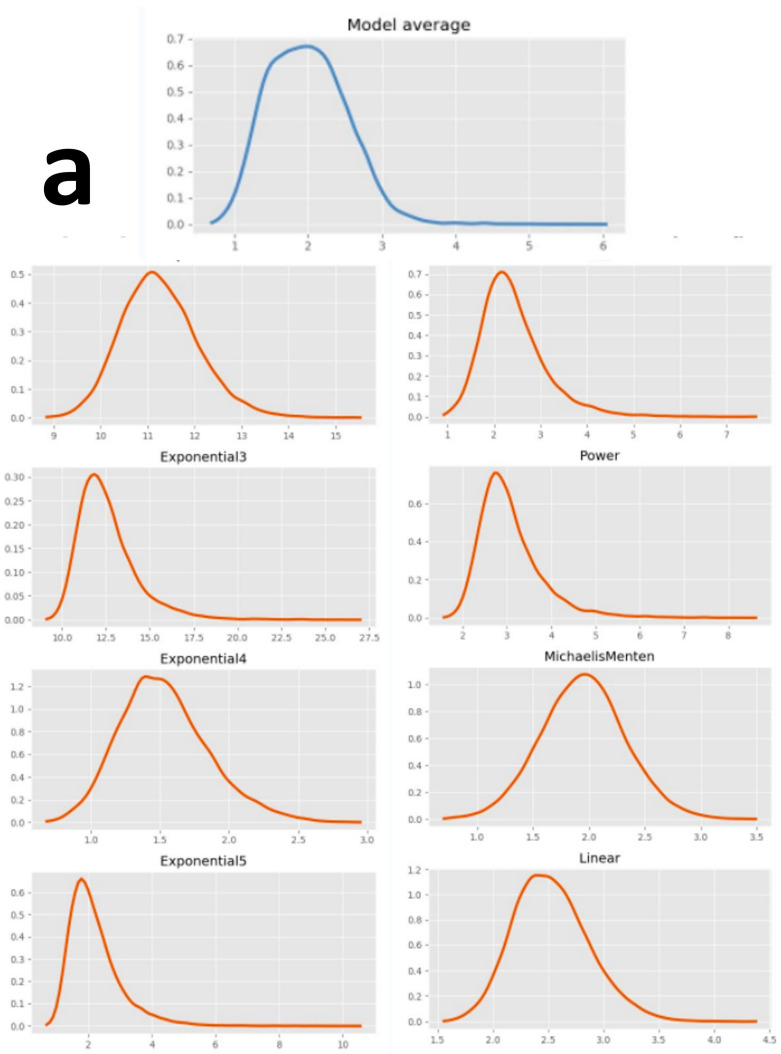

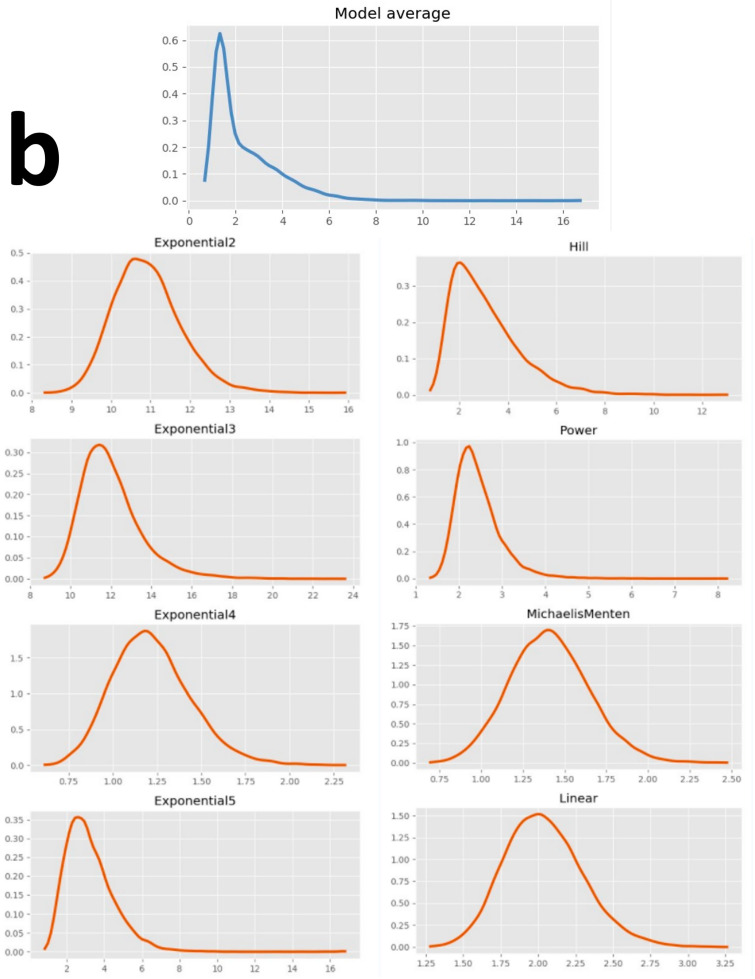

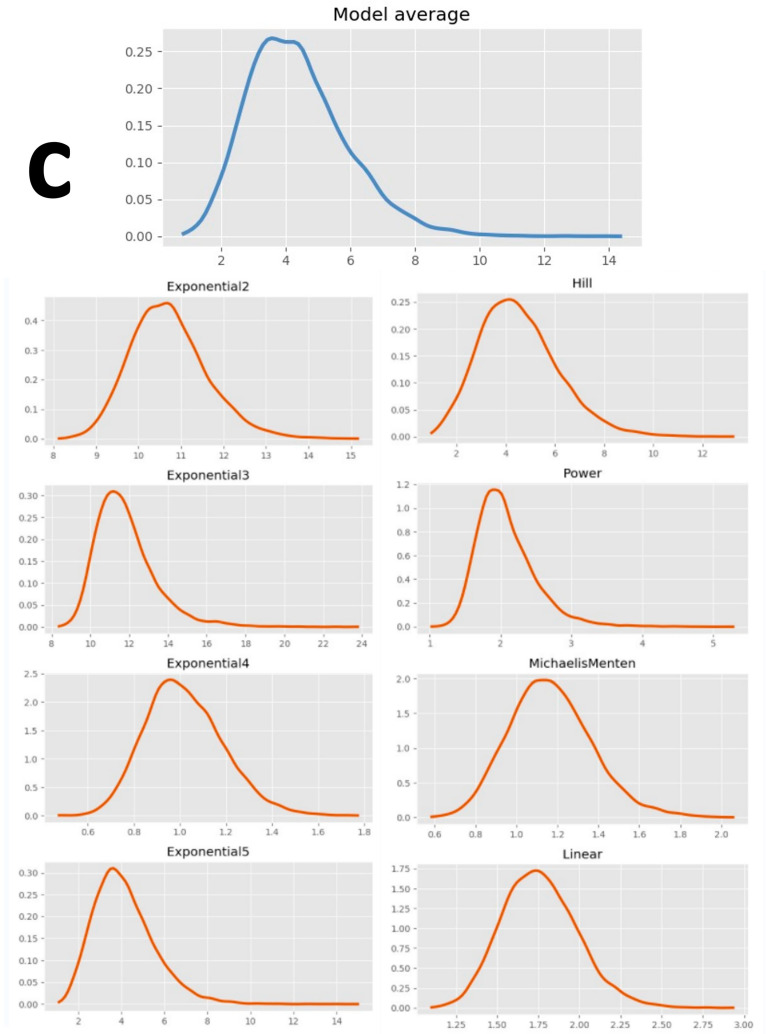

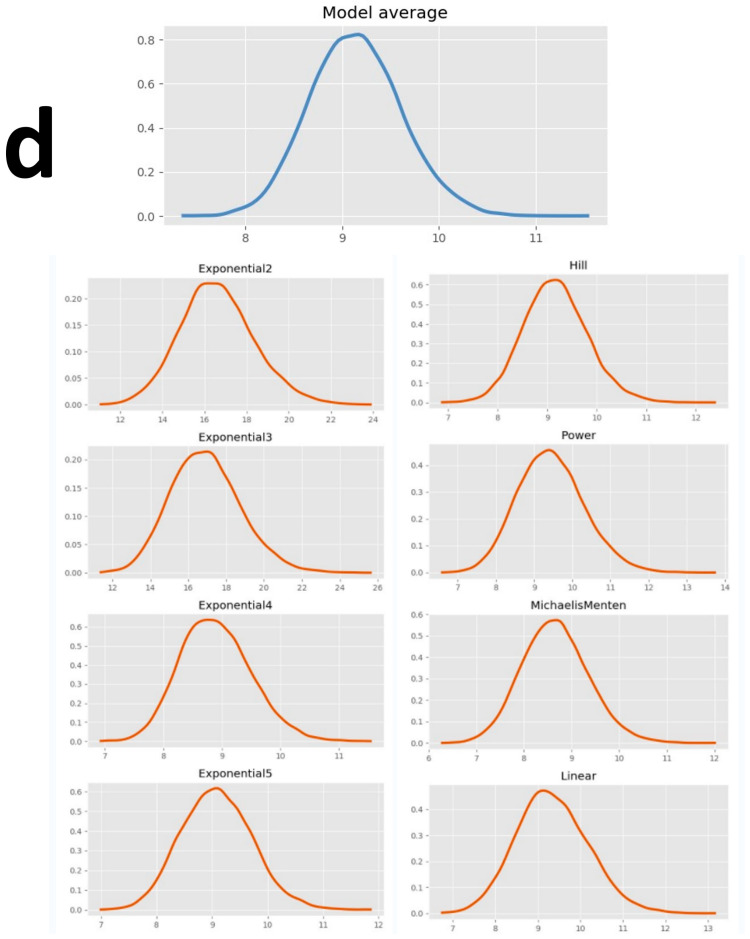

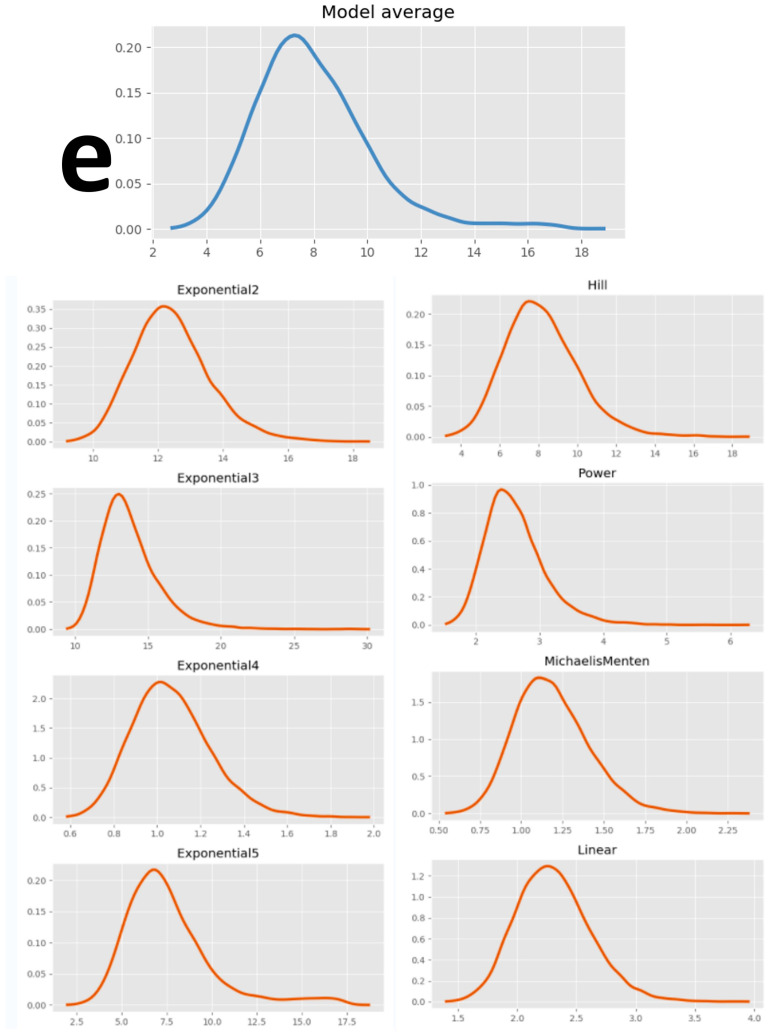

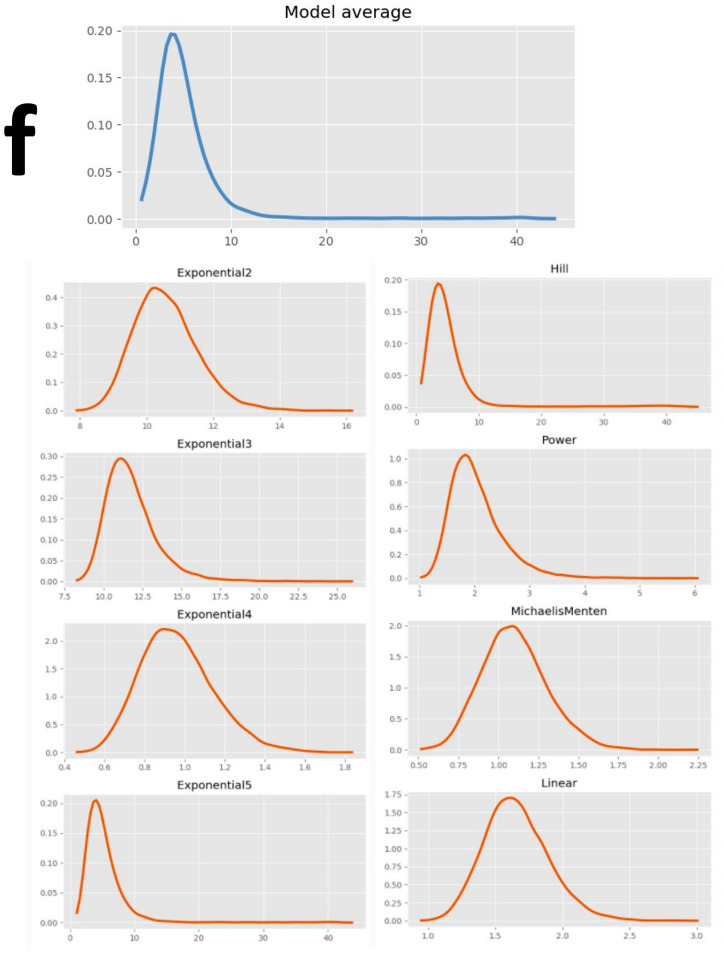
Model average and the eight BMD models of BMC estimates in BMR = 10%. (**a**–**e**) CM at 1–5 dpf; (**f**) CMA at 5 dpf.

Based on the BBMD analysis, the posterior predictive *p*-value for model fit (PPP values) ranged from 0.05 to 0.95, indicating that all models adequately fit the data. The majority of $\hat{r}$ values suggests that the MCMC sampling converged well. The results revealed an appropriate dose–response relationship between W-F concentration and toxicological indicators in the eight standard BMD models, implying a correlation between them (Supplementary Figures S1 and S2). The LC_50_/EC_50_ are presented in Table 4. Table 5 displays the estimated individual models and model-averaged BMCs for a 10% increase in risk for toxicity indicators (BMR_10_). As the BMR increased, both the BMCs/BMDLs also increased (Figure 4).

## 4. Discussion

The toxicity assessment of environmental pollutants should encompass acute, short-term, subchronic, reproductive, developmental, and chronic effects to ascertain the types and degrees of adverse health impacts that these pollutants may exert [61]. Prior to evaluation, it is essential to consult the toxicity database to gather fundamental toxicity data. Acute and short-term toxicity tests were conducted using zebrafish embryos and yolk sac larvae, which provide essential data, including LC_50_, NOAEL, and LOAEL. These data serve as critical support for longer-term toxicity experiments and informs future updates to relevant water quality standards [47]. Previous studies investigating acute and short-term toxicological effects of fluoride exposure during the early life stage of aquatic fish have reported varying concentrations, ranging from 51 mg/L to 1045.8 mg/L [24,25,50,51,54]. This variability can primarily be attributed to the use of different species of experimental fish, including zebrafish, rainbow trout, blackhead catfish, and peacock fish, as well as variations in exposure duration, which range from 24 h to extended periods. Additionally, fluoride’s capacity to interact with calcium ions in water to form precipitates leads to a reduction in fluoride concentration, thereby establishing a positive correlation between the LC_50_ of fluoride and water hardness [62]. Consequently, differences in calcium ion concentration across previous studies have also contributed to the observed variations in LC_50_ results. To obtain fundamental data, this study conducted tests using zebrafish from 2 hpf to 5 dpf, calculating the LC_50_/EC_50_ from 1 to 5 dpf. Moreover, E3 water was used to prepare a fluoride exposure solution to mitigate the influence of calcium ions in the water. The results indicated a dose–response relationship between fluoride concentration in the water and CM rates at 1, 2, 3, 4, and 5 dpf, as well as the CMA rate at 5 dpf in early-life-stage zebrafish. Additionally, there were no statistically significant differences in the primary toxicity observation indicators between the fluoride exposure groups at the ‘ESLC’ and the control group. In this study, the concentration of fluoride at 20 mg/L is highlighted as the LOAEL for most toxicity indicators. It is important to note that there are numerous areas in natural water environments where concentrations exceed 20 mg/L. For instance, a survey revealed that the highest fluoride concentration in surface water and groundwater in 16 major cities in Pakistan was 24.48 mg/L [61], while in the Naivasha Basin of Kenya, groundwater reached levels of 43.6 mg/L [62]. Furthermore, lakes and basins in central Ethiopia showed fluoride concentrations as high as 68.9 mg/L [63]. These findings serve as a reminder of the importance of considering the toxic risks of fluoride in natural aquatic environments on aquatic ecosystems. It is essential for academia and policymakers to work together in developing comprehensive solutions to mitigate and decrease fluoride concentrations in water environments.

In our research, the BMCs and BMCLs were analyzed using the BBMD system [58] based on BMA. Addressing the ongoing challenge of variable selection for risk factor modeling in statistical practice, BMA considers all models with non-negligible probabilities and summarizes the posterior probabilities for all variables at the end, leading to more reliable and robust effect estimates [64,65,66,67]. BMA BMC has been widely utilized in various fields [68,69,70,71,72]. In our study, the BMCL10 for W-F exposure in zebrafish embryos and sac–fry stage ranged from 1.02 to 4.98 mg/L. These results were calculated based on a benchmark response of 10 (BMR = 10). The BMR represents a level of response in a specific endpoint that is measurable, considered relevant to humans or model species, and is used to estimate the associated dose (the ‘true’ BMD) [45]. For quantal data, the BMR is defined as an increase in the incidence of the lesion/response scored compared to the background incidence [45]. Previous guidance from the EFSA Scientific Committee (EFSA SC) on BMD modeling indicated that several studies estimated that the median of the upper bounds of extra risk at the NOAEL was around 10%, implying that the BMDL10 might be suitable in many instances [73,74,75]. In this study, it was found that when the fluoride concentration in water exceeded 1.02 mg/L, there was a 10% increased risk of mortality for zebrafish embryos. When an external source of mortality impacts a population, it can affect the number of individuals or the total biomass in a particular stage [76,77,78,79,80,81]. Population fluctuations can influence species diversity and have significant consequences for ecosystems [82]. Additionally, many developmental abnormalities can be attributed to mutations in genes that encode enzymes and structural proteins [83]. Genomic alterations and mutations are recognized as hallmark insults resulting from environmental chemicals [84]. Currently, we are conducting a study on the effects of transcriptomics during the embryonic and sac–fry stages of zebrafish exposed to ‘ESLC’ of W-F. Therefore, based on the three dimensions of biodiversity—gene diversity, species diversity, and ecosystem diversity—we recommend calculating the BMCs and BMCLs of W-F, from the genetic to the species level, to separately assess toxicity risk in the future. This approach will contribute to the protection of biodiversity and promote the sustainable development of the ecological environment.

When establishing safety limits for chemicals, toxicological data obtained from animal experiments serve as a critical reference point. The selection of safety coefficients and uncertainty factors is significantly influenced by species and individual differences among experimental animals, making these data the most important in computational toxicology research when extrapolating results from animal models to humans. Currently, data from mammalian experiments are predominantly utilized, and a tenfold uncertainty factor (UF) is commonly applied in the derivation process [85]. However, due to cost and ethical considerations, there are inherent limitations to the use of mammals in toxicology experiments [86]. In contrast, zebrafish models align more closely with the 4R principles of reduction, refinement, substitution, and responsibility [87,88]. Consequently, extrapolating research results from zebrafish to humans presents not only a significant challenge in computational toxicology but also represents a prominent topic and future direction for development. With the ongoing advancement of technology, progress has been made in this field. An invention patent titled “Conversion Method of Zebrafish to Human Dose for Safety Evaluation” (patent number: ZL2020 10256136.8) was approved by China Huante Biotechnology Co., Ltd. in 2020, serving as an example [31]. The patent proposes an approach for converting acute and short-term toxicity test data from zebrafish to mammals, followed by the extrapolation of these concentrations to humans. The specific calculation method is (1) UFs_zebrafish_ = UFs_mammals_ ÷ 10Average(Log LC_50zebrafish_/Log LC_50mammals_); (2) HBGV_humans_ = NOAEL_humans_ ÷ UFs_zebrafish_. Although this represents only the beginning, advancements in technology and the emergence of novel methodologies will enhance the feasibility of extrapolating human data using findings from zebrafish. The foundational toxicological data obtained from zebrafish at the early life stage in this study, which includes BMC, BMCL, LC_50_, and EC_50_ values in response to fluoride exposure, lays the groundwork for future research.

There are several limitations to our research. Firstly, although the zebrafish used in this study is a standard model animal for environmental chemical toxicity risk assessment recommended by the OECD [4], it cannot fully represent the diversity of fish species in different water regions. Secondly, variations in calcium ion concentrations across different water bodies need to be taken into account when assessing the toxicity risks of fluoride ions [89]. Toxicity calculations should be conducted separately based on the specific calcium concentrations in water. The E3 water utilized in this study has low calcium levels [47]; thus, our research only provides baseline BMCs and BMCLs. Thirdly, the OECD emphasizes the importance of testing chemicals throughout the entire fish life cycle to accurately estimate aquatic ecotoxicity risks [4]. Additionally, it is important to note that the results of this study cannot be directly extrapolated to humans at this stage. Future research should consider incorporating population epidemiology studies for a more comprehensive understanding [90].

## 5. Conclusions

**Figure 5:**
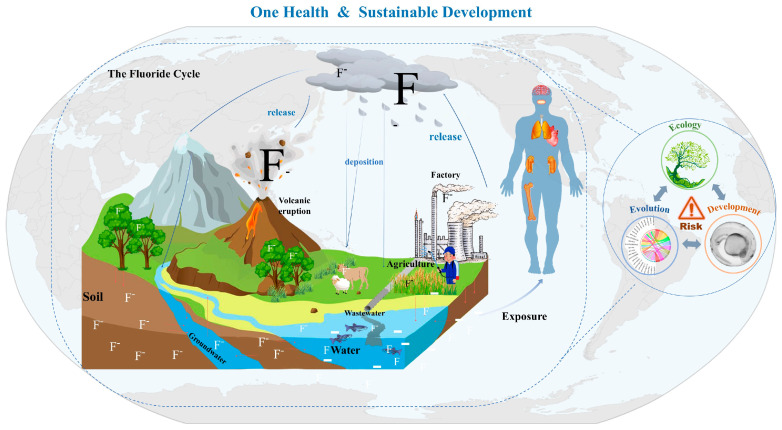
One health conception in fluoride for sustainability development of the environment. F^-^ represents fluoride ions.

Our study indicates that the range of BMCL10 for W-F exposure during the zebrafish’s early life is between 1.02 and 4.98 mg/L. The basic toxicity data for the early life stages of zebrafish, obtained through BMA BMD calculations, are expected to inform future research aimed at promoting sustainable environmental development. To this end, we propose the concept of ‘One Health of Fluoride’ (Figure 5), which advocates for a comprehensive approach that encompasses environmental, animal, and human health while establishing the W-F guideline based on the Eco Evo Devo framework [91,92]. This concept underscores the significance of social factors, encourages interdisciplinary collaboration, and aims to mitigate the adverse effects of fluoride on the environment, improve equitable health outcomes, preserve biodiversity, and lay a foundation for sustainability.

## Figures and Tables

**Table 1:** The formulas for toxicity indicators estimate.

Toxicity Indicators	Formula
CM	N = cumulative dead numbers each day/total
CMA at 5 dpf	N = CMA numbers from hatch to 5 dpf/hatched numbers

**Table 2:** The models for dose–response and BMC/BMCL estimates.

Model	Formula
Exponential model 2	$f\left(dose\right)=a\times e^{b\times dose}$
Exponential model 3	$f\left(dose\right)=a\times e^{b\times {dose}^{g}}$
Exponential model 4	$f\left(dose\right)=a\times \left[c-\left(c-1\right)\times e^{-b\;\times \;dose}\right]$
Exponential model 5	$f\left(dose\right)=a\times \left[c-\left(c-1\right)\times e^{-{\left(b\;\times \;dose\right)}^{g}}\right]$
Hill model	$f\left(dose\right)=a+\frac{b\times {dose}^{g}}{c^{g}+{dose}^{g}}$
Power mode	$f\left(dose\right)=a+b\times {dose}^{g}$
Michaelis Menten model	$f\left(dose\right)=a+\frac{b\times dose}{c+dose}$
Linear model	$f\left(dose\right)=a+b\times dose$

**Table 3:** The outcome of toxicity indicators for each concentration group ($\bar{x}$ ± SD).

Fluoride Dose (mg/L)	CM at					CMA at 5 dpf
	1 dpf	2 dpf	3 dpf	4 dpf	5 dpf	
0.0	6.11 ± 2.36	10.67 ± 3.82	10.67 ± 3.82	10.67 ± 3.82	13.89 ± 3.28	9.45 ± 3.10
0.5	8.56 ± 4.45	10.89 ± 3.54	10.89 ± 3.54	10.89 ± 3.54	14.78 ± 3.99	10.66 ± 5.38
1.0	8.00 ± 4.56	9.33 ± 3.50	9.33 ± 3.50	9.33 ± 3.50	14.56 ± 4.39	10.56 ± 8.43
4.0	10.00 ± 9.78	10.56 ± 9.78	10.56 ± 9.78	10.56 ± 9.78	12.22 ± 10.30	8.55 ± 8.49
10.0	13.00 ± 8.99	13.17 ± 8.73	13.17 ± 8.73	14.17 ± 8.33	16.83 ± 8.52	16.98 ± 4.34
20.0	23.93 ± 6.27	24.93 ± 7.74	25.43 ± 8.21	26.26 ± 7.72	27.93 ± 11.10	24.86 ± 9.23
50.0	24.44 ± 7.79	51.11 ± 24.10	51.11 ± 24.10	60.56 ± 22.75	66.67 ± 16.73	27.46 ± 10.06
80.0	35.00 ± 10.90	51.11 ± 16.29	57.78 ± 22.77	78.33 ± 19.41	85.56 ± 7.50	87.24 ± 12.26
100.0	33.89 ± 7.13	61.11 ± 29.34	85.00 ± 17.98	97.22 ± 3.90	100.00 ± 0	100 ± 0
120.0	46.11 ± 2.51	76.11 ± 15.41	83.89 ± 14.21	90.00 ± 10.75	100.00 ± 0	90.00 ± 10.75
150.0	42.78 ± 9.29	76.67 ± 22.11	98.33 ± 4.08	100.00 ± 0	100.00 ± 0	100.00 ± 0
200.0	54.45 ± 16.82	98.33 ± 2.79	99.45 ± 1.36	100.00 ± 0	100.00 ± 0	100.00 ± 0
250.0	66.67 ± 5.27	100.00 ± 0	100.00 ± 0	100.00 ± 0	100.00 ± 0	100.00 ± 0
300.0	100.00 ± 0	100.00 ± 0	100.00 ± 0	100.00 ± 0	100.00 ± 0	100.00 ± 0

**Table 4:** The LC_50_/EC_50_ of toxicity indicators on embryo and sac–fry stages of zebrafish.

Model	1 dpf CM			2 dpf CM			3 dpf CM			4 dpf CM			5 dpf CM			5 dpf CMA		
	Rhat	PPPv	LC_50_	Rhat	PPPv	LC_50_	Rhat	PPPv	LC_50_	Rhat	PPPv	LC_50_	Rhat	PPPv	LC_50_	Rhat	PPPv	EC_50_
Exponential model 2	1.0	0.259	186.00	1.0	0.263	142	1.0	0.267	135	1.0	0.262	135	1.0	0.260	114	1.0	0.266	132
Exponential model 3	1.0	0.268	186.00	1.0	0.259	143	1.0	0.263	134	1.0	0.261	134	1.0	0.263	115	1.0	0.265	134
Exponential model 4	1.0	0.266	147.00	1.0	0.261	75	1.0	0.261	63	1.0	0.265	63	1.0	0.260	45	1.0	0.260	60
Exponential model 5	1.0	0.269	143.00	1.0	0.259	70	1.0	0.272	57	1.0	0.265	57	1.0	0.259	38	1.0	0.263	56
Hill model	1.0	0.254	145.00	1.0	0.260	74	1.0	0.267	56	1.0	0.264	56	1.0	0.264	37	1.0	0.265	58
Michaelis Menten model	1.0	0.263	150.00	1.0	0.259	93	1.0	0.266	82	1.0	0.266	82	1.0	0.266	69	1.0	0.258	79
Linear model	1.0	0.271	148.00	1.0	0.256	81	1.0	0.269	69	1.0	0.265	69	1.0	0.267	50	1.0	0.258	65
Power model	1.0	0.268	149.00	1.0	0.258	92	1.0	0.262	81	1.0	0.264	81	1.0	0.263	67	1.0	0.259	77
Average	\	\	147.00	\	\	80.80	\	\	61.25	\	\	56.50	\	\	37.50	\	\	59.75

**Table 5:** W-F BMCs/BMCLs (mg/L) for the zebrafish on embryo and sac–fry stages based on BMRs of 10% reduction in toxicity indicators.

Model	1 dpf CM				2 dpf CM				3 dpf CM				4 dpf CM				5 dpf CM				5 dpf CMA			
	Weight (%)	BMC	BMCL	BMCU	Weight (%)	BMC	BMCL	BMCU	Weight (%)	BMC	BMCL	BMCU	Weight (%)	BMC	BMCL	BMCU	Weight (%)	BMC	BMCL	BMCU	Weight (%)	BMC	BMCL	BMCU
Model average	\	1.96	1.19	2.88	\	1.80	1.02	5.07	\	4.19	2.19	7.19	\	5.07	3.06	7.87	\	7.69	4.98	11.82	\	3.73	1.69	7.82
Exponential model 2	0.00	11.20	10.03	12.69	0.00	10.85	9.66	12.41	0.00	10.64	9.36	12.33	0.00	10.82	9.40	12.72	0.00	12.32	10.67	14.59	0.00	10.31	8.96	12.13
Exponential model 3	0.00	12.26	10.54	15.85	0.00	11.76	10.08	14.87	0.00	11.56	9.86	14.67	0.00	11.81	9.93	15.18	0.00	13.42	11.24	17.43	0.00	11.27	9.50	14.65
Exponential model 4	27.40	1.50	1.05	2.12	23.10	1.20	0.89	1.61	1.70	1.01	0.76	1.32	0.00	0.81	0.62	1.05	0.00	1.05	0.80	1.39	3.70	0.87	0.64	1.17
Exponential model 5	4.60	2.04	1.27	3.80	24.60	3.01	1.59	5.60	45.60	3.95	2.18	6.74	0.40	4.54	2.75	7.26	0.41	7.12	4.56	12.67	62.20	3.88	1.98	8.04
Hill model	6.40	2.31	1.51	3.74	28.60	2.77	1.44	5.77	51.50	4.37	2.20	7.44	0.60	5.34	3.32	8.02	0.59	8.00	5.40	11.53	29.80	3.30	1.36	6.91
Michaelis Menten model	33.10	1.95	1.33	2.58	23.30	1.40	1.03	1.82	1.10	1.15	0.85	1.52	0.00	0.89	0.65	1.21	0.00	1.17	0.86	1.59	4.20	0.99	0.71	1.33
Linear model	24.70	2.51	2.01	3.14	0.20	2.02	1.64	2.50	0.00	1.75	1.41	2.16	0.00	1.59	1.28	1.99	0.00	2.29	1.84	2.85	0.00	1.52	1.19	1.93
Power model	3.80	2.93	2.21	4.39	0.30	2.35	1.80	3.41	0.00	2.01	1.54	2.83	0.00	1.79	1.37	2.47	0.00	2.55	1.98	3.50	0.00	1.79	1.33	2.64

## Data Availability

The original contributions presented in this study are included in the article/Supplementary Material. Further inquiries can be directed to the corresponding authors.

## Supplementary Materials

The following supporting information can be downloaded at: https://www.mdpi.com/article/10.3390/toxics12120902/s1, Figure S1: Linear fit summary. The dose-response relationship between the W-F concentration (mg/L) and CM; Figure S2: Linear fit summary. The dose-response relationship between the W-F concentration (mg/L) and CMA at 5 dpf.

## Abbreviations

BBMD	Bayesian benchmark dose analysis system.
BMA	Bayesian model averaging.
BMC	benchmark concentration.
BMCL	lower bound of the credible interval of benchmark concentration.
BMD	benchmark dose.
BMDL	lower bound of the credible interval of benchmark dose.
BMR	benchmark response.
CM	cumulative mortality.
CMA	cumulative malformation rate.
dpf	days post-fertilization.
EC50	effect concentration for 50% effect.
EFSA	European Food Safety Authority.
EFSA SC	EFSA Scientific Committee.
EPA	U.S. environmental protection agency.
ESLC	environmental standard limit concentration.
hpf	hours post-fertilization.
ICs	internal plate controls.
LC50	median lethal dose.
LOAEL	lowest observed adverse effect level.
LSD	least significant difference.
NaF	sodium fluoride.
NOAEL	no observed adverse effect level.
OECD	Organization for Economic Cooperation and Development.
SD	standard deviation.
UFs	uncertainty factors.
W-F	water fluoride.
WHO	World health organization.
