# Supplementary material for: Analysis of Acute and Short-Term Fluoride Toxicity in Zebrafish Embryo and Sac–Fry Stages Based on Bayesian Model Averaging
Source: Toxics. 2024 Dec 11;12(12):902. doi: 10.3390/toxics12120902 (PMC11728822; doi:10.3390/toxics12120902)
Supplement: Supplementary file 1 [file toxics-12-00902-s001.zip › toxics-3339401-supplementary.pdf]

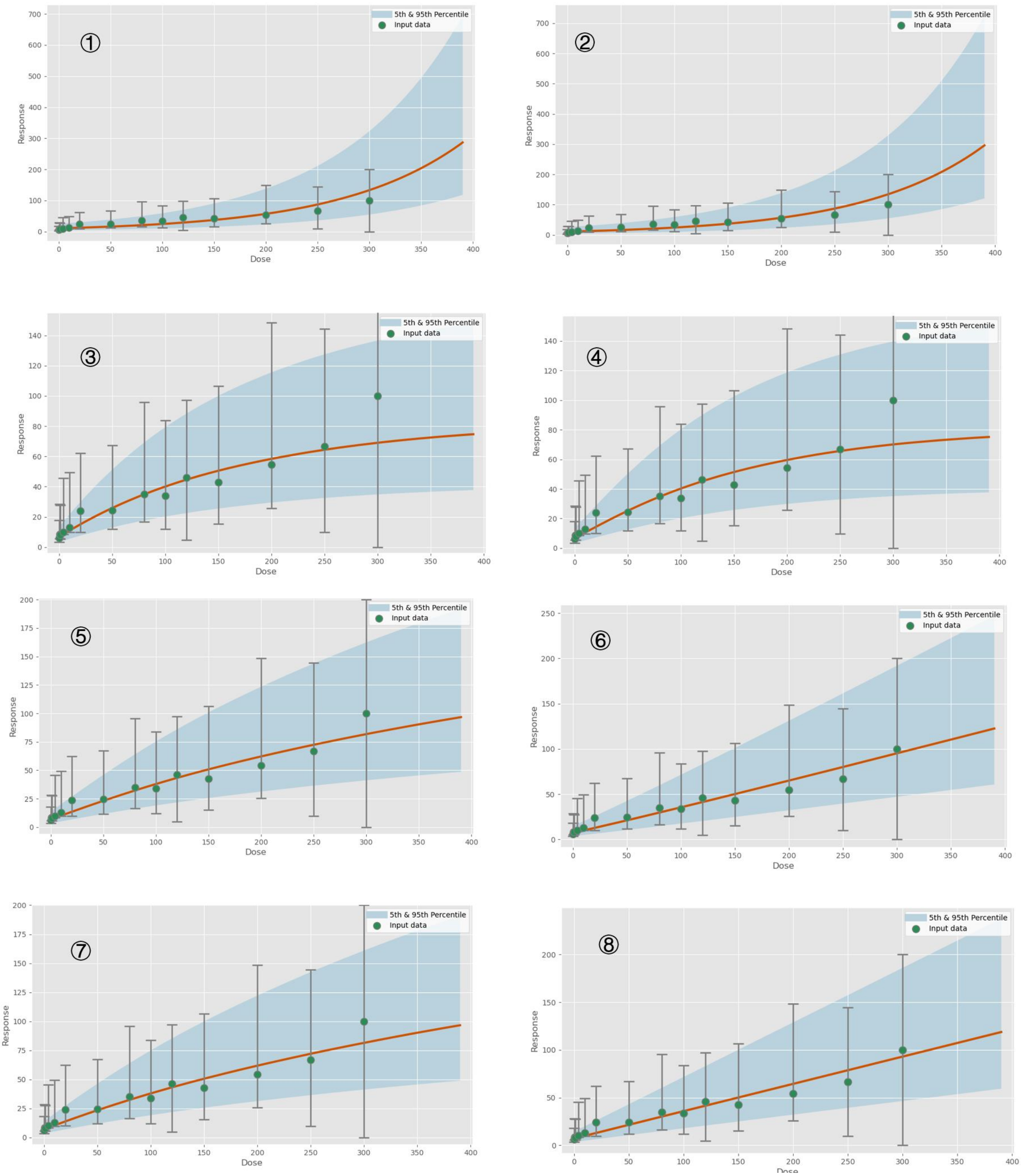
①
②
③
④
⑤
⑥
⑦
⑧
5th & 95th Percentile
Input data
Response
Dose

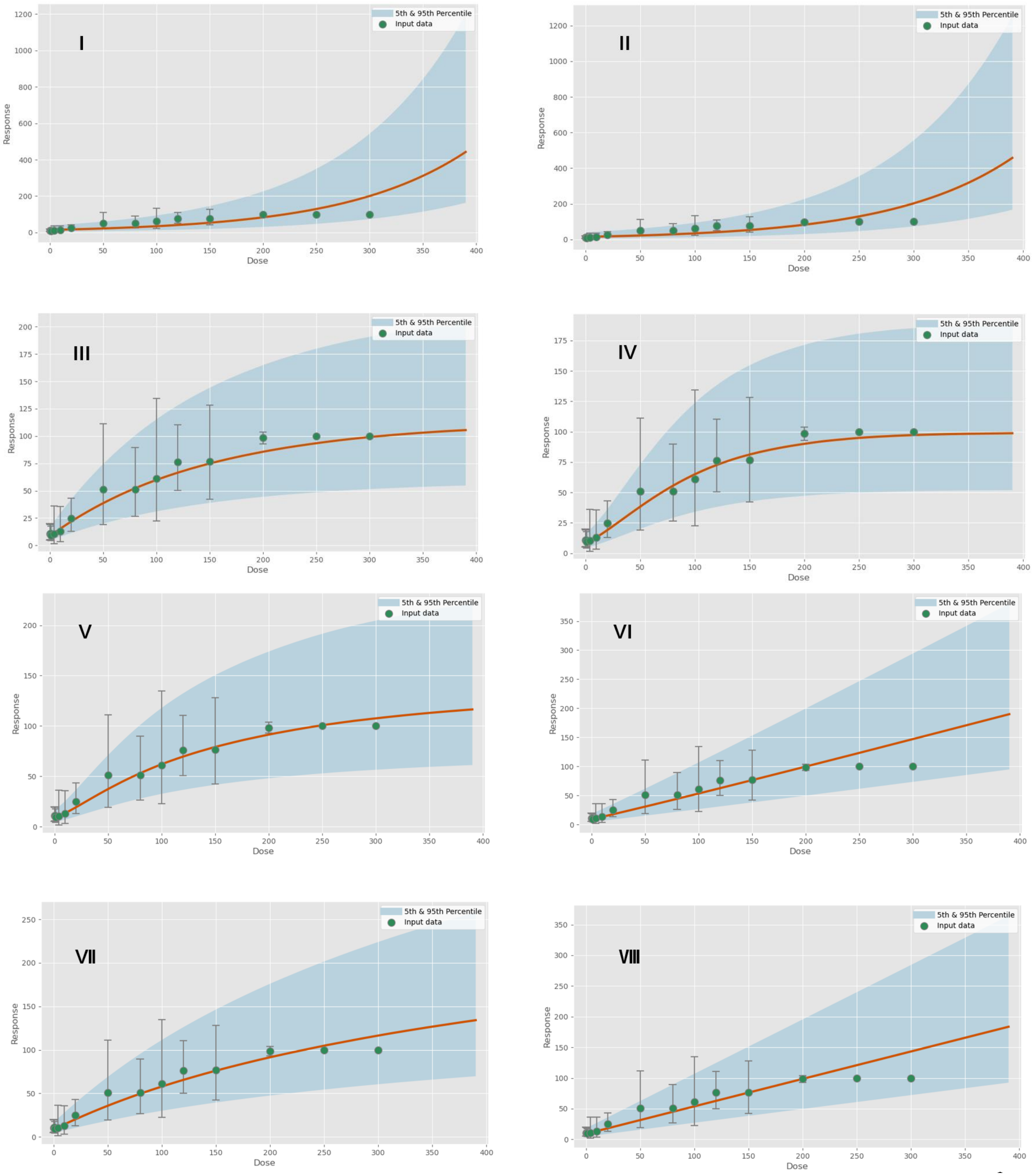
I
II
III
IV
V
VI
VII
VIII
5th & 95th Percentile
Input data
Response
Dose

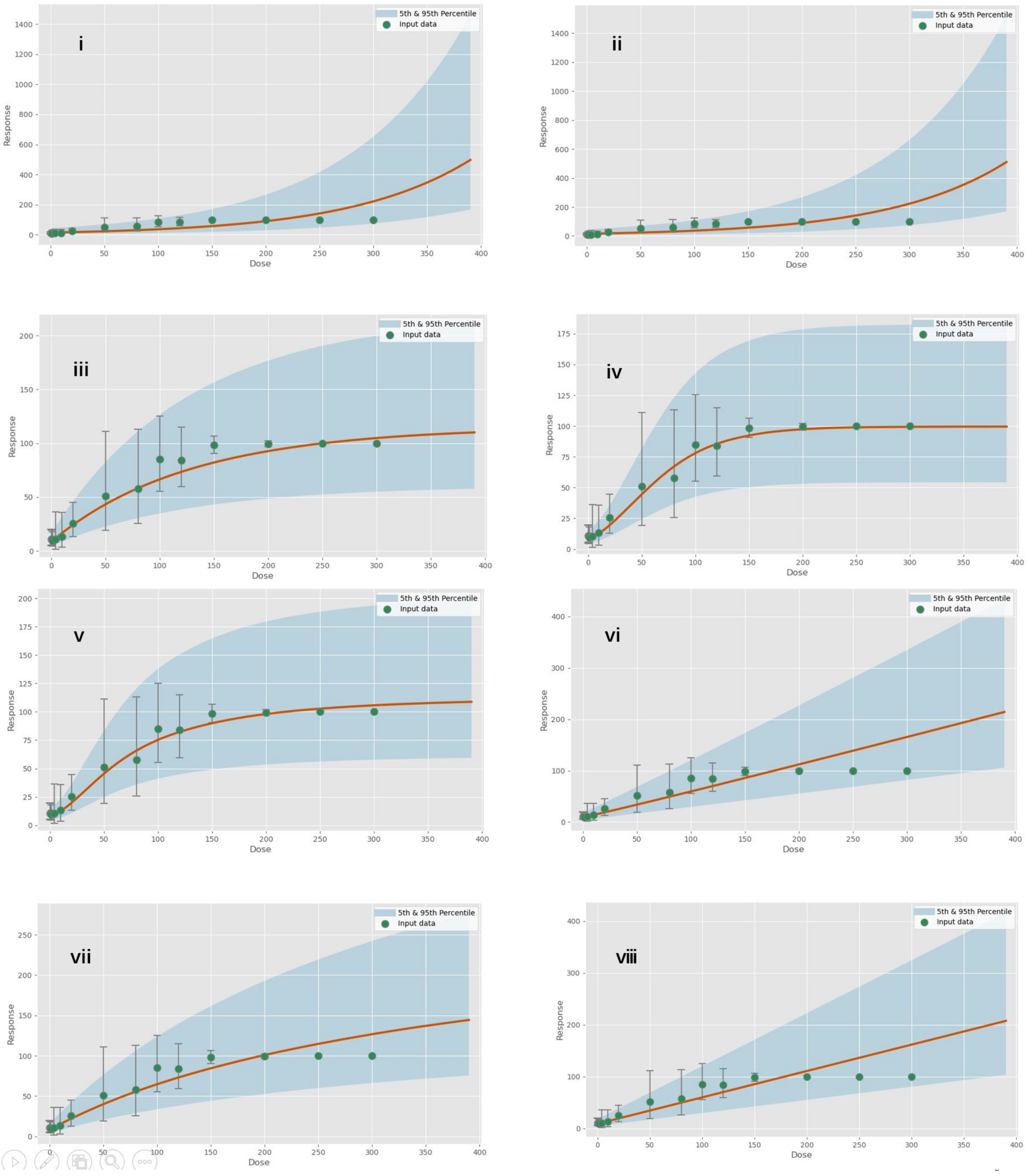
i
ii
iii
iv
v
vi
vii
viii
5th & 95th Percentile
Input data
Response
Dose

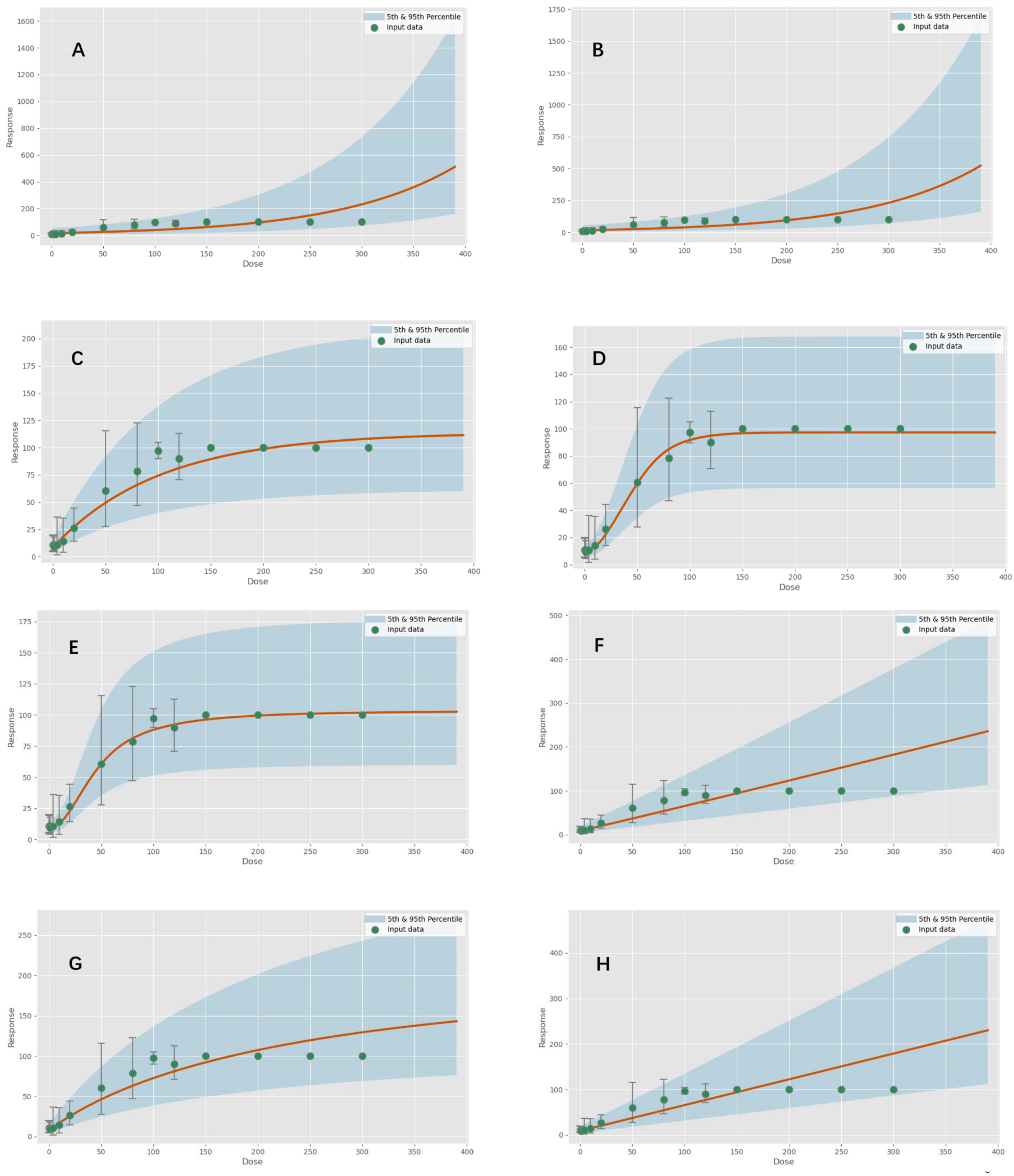
A
B
C
D
E
F
G
H
5th & 95th Percentile
Input data
Response
Dose

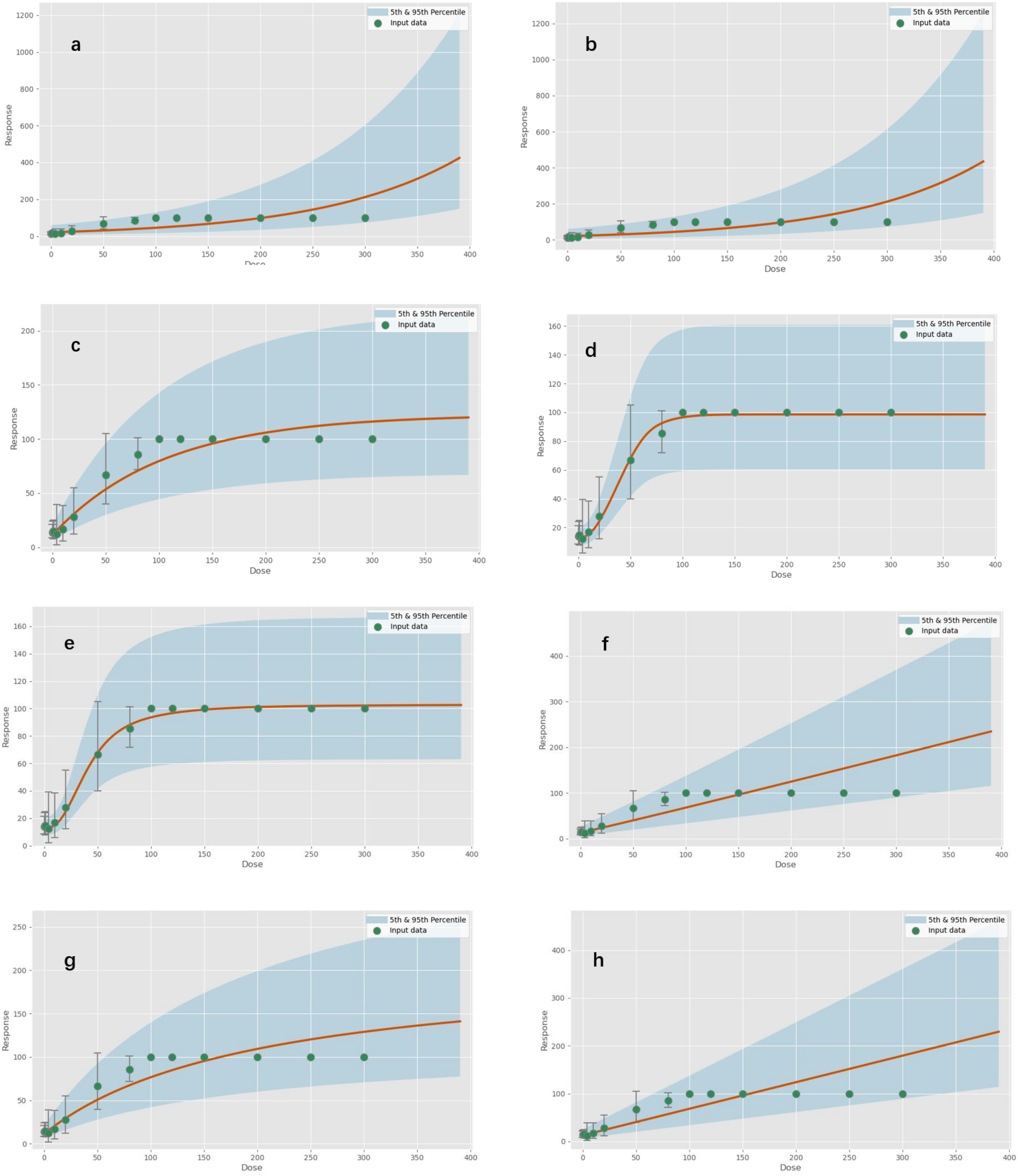

Supplementary Figure S1. Linear fit summary. The dose-response relationship between the W-F concentration (mg/L) and CM at 1 dpf (①-⑧), 2 dpf (Ⅰ-Ⅷ), 3 dpf (ⅰ-ⅷ), 4 dpf (A-H) and 5 dpf (a-h) in the eight BMD models was appropriate. (①, Ⅰ, ⅰ, A, **a**) were Exponential model 2; (②, Ⅱ, ⅱ, B, **b**) were Exponential model 3; (③, Ⅲ, ⅲ, C, **c**) were Exponential model 4; (

④， Ⅳ， iv， **D**， **d**) were Exponential model 5; (⑤， Ⅴ， v， **E**， **e**) were Hill model; (⑥， Ⅵ， vi， **F**， **f**) were Power model; (⑦， Ⅶ， vii， **G**， **g**) were Michaelis–Menten model; (⑧， Ⅷ， viii， **H**， **h**) were Linear model.

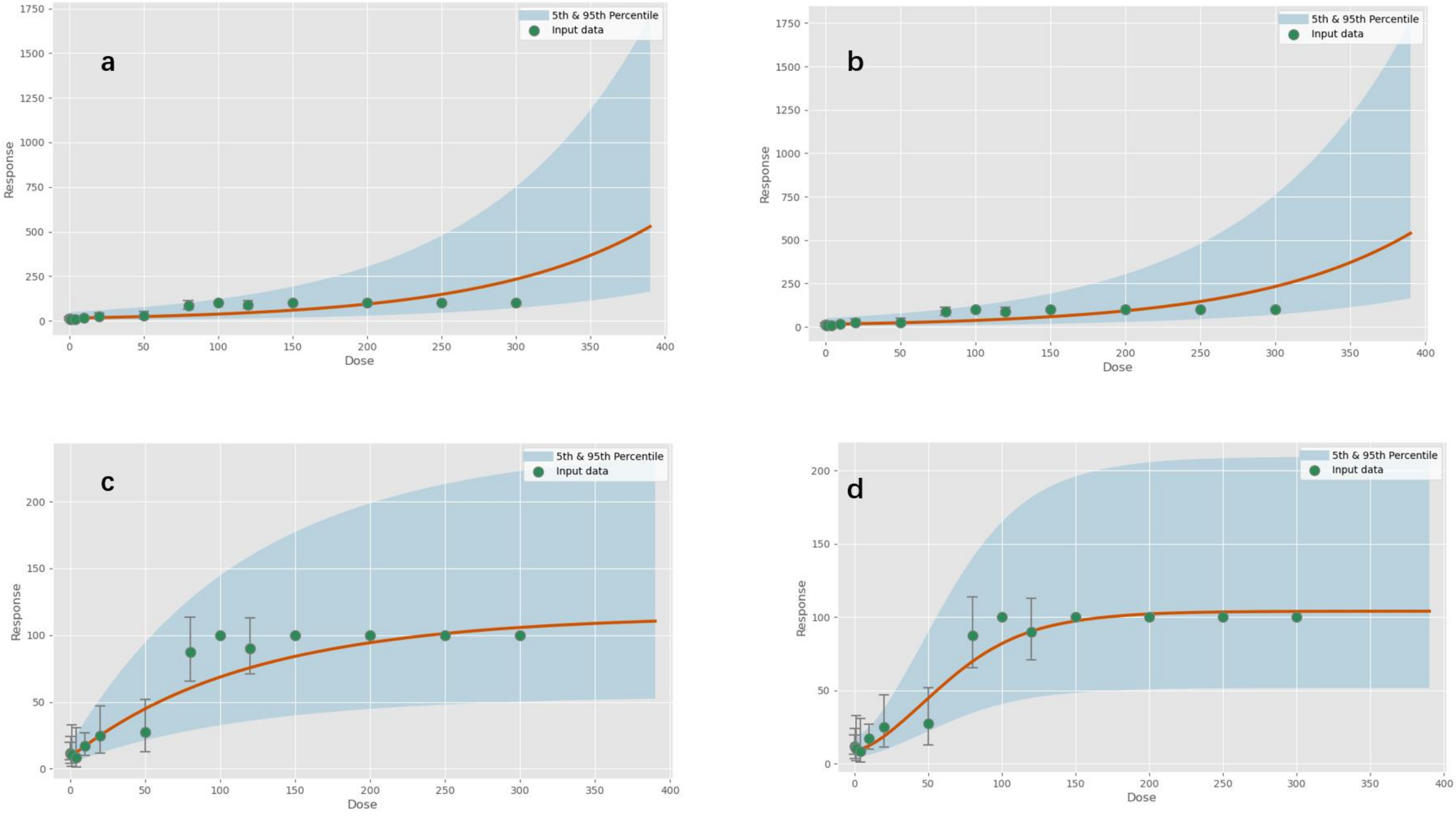

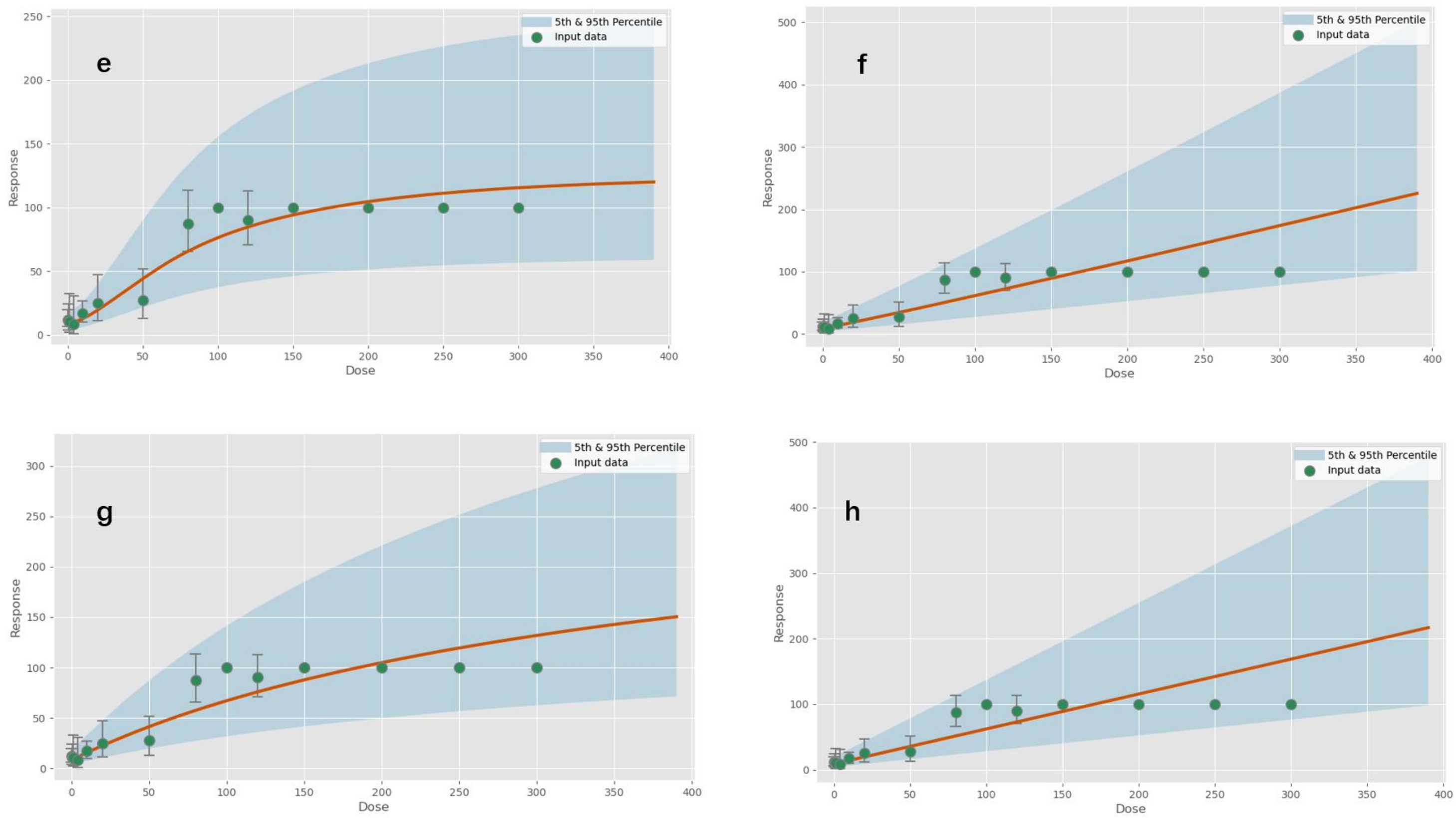

Supplementary Figure S2. Linear fit summary. The dose-response relationship between the W-F concentration (mg/L) and CMA at 5 dpf in the eight BMD models was appropriate. (a) Exponential model 2; (b) Exponential model 3; (c) Exponential model 4; (d) Exponential model 5; (e) Hill model; (f) Power model; (g) Michaelis–Menten model; (h) Linear mode.
